# Linking migratory performance to breeding phenology and productivity in an Afro-Palearctic long-distance migrant

**DOI:** 10.1038/s41598-021-01734-0

**Published:** 2021-12-01

**Authors:** Joana S. Costa, Steffen Hahn, Pedro M. Araújo, Kiran L. Dhanjal-Adams, Afonso D. Rocha, José A. Alves

**Affiliations:** 1 CESAM - Centre for Environmental and Marine Studies, Department of Biology, University of Aveiro, Aveiro, Portugal; 2 Department of Bird Migration, Swiss Ornithological Institute, Sempach, Switzerland; 3 CIBIO/InBIO, Centro de Investigação em Biodiversidade e Recursos Genéticos, Campus Agrário de Vairão, Universidade Do Porto, Vairão, Portugal; 4 MARE – Marine and Environmental Sciences Centre, Department of Life Sciences, University of Coimbra, Coimbra, Portugal; 5 Centre for the Advanced Study of Collective Behaviour, University of Konstanz, Konstanz, Germany; 6 Ecology of Animal Societies, Max Planck Institute of Animal Behavior, Konstanz, Germany; 7 South Iceland Research Centre, University of Iceland, Laugarvatn, Iceland

**Keywords:** Animal migration, Population dynamics

## Abstract

Understanding the relationship between migratory performance and fitness is crucial for predicting population dynamics of migratory species. In this study, we used geolocators to explore migration performance (speed and duration of migratory movements, migratory timings) and its association with breeding phenology and productivity in an Afro-Palearctic insectivore, the European bee-eater (*Merops apiaster*), breeding in Iberian Peninsula. Bee-eaters migrated at higher travel speeds and had shorter travel duration in spring compared to autumn. Individuals that departed earlier or spent fewer days in-flight arrived earlier to the breeding areas. Our results show overall positive, but year-specific, linkages between arrival and laying dates. In one year, laying was earlier and productivity was higher, remaining constant throughout the season, while in the subsequent year productivity was lower and, importantly, declined with laying date. These results suggest that arriving earlier can be advantageous for bee-eaters, as in years when breeding conditions are favourable, early and late breeders produce high and similar number of fledglings, but when conditions are unfavourable only early breeders experience high productivity levels.

## Introduction

Population declines are currently widespread in migratory birds across the world^1,2^. Determining when (in the annual cycle) and where (across the distribution range) migrants may be constrained and how this impacts demography (e.g. population change), requires a detailed understanding of individual performance in time and space, throughout the annual cycle^3–5^. In the Afro-Palaearctic migratory system population declines have been reported since the 1970s^6^, and these negative trends are of increasing concern^1,7^. However, exploring the potential factors affecting long-distance migrants is particularly challenging, as conditions at very distant locations experienced in a given season, may also affect individual performance in a subsequent stage of the annual cycle at another location (i.e. carry-over effects^8^).

Migratory birds must time annual cycle events to coincide with local environmental conditions at all stages of the annual cycle if they are to maximize fitness (i.e., survival and reproductive success), despite the difficulty of predicting conditions at breeding/wintering sites hundreds of kilometres away. Timing of spring migration is frequently associated with breeding success, making it a key stage of the annual cycle, linking behaviour with fitness. Indeed, early arrival to the breeding areas may be advantageous, as it increases the likelihood of encountering favourable environmental conditions (e.g. high food availability^9^), of securing high quality habitats, and of allowing re-nesting following potential nest failure^10,11^. Furthermore, for many species, spring migration is shorter and/or faster than autumn migration^12^ as individuals are under high selection pressure to arrive earlier. However, early arrival and associated benefits may only be possible to those individuals that migrate shorter distances^13^ or are able to initiate migration earlier and/or travel faster^14–16^.

The distance between breeding and non-breeding areas often determines the time spent on migration and therefore the timing of spring arrival^13,17^. The quality of habitat during the non-breeding season can also play an important role in dictating migratory timing and subsequent breeding performance^18,19^. Individuals occupying high quality habitats during the non-breeding season may accumulate fuel at a fast rate and initiate spring migration in good condition^20^, thus increasing the probability of raising more offspring in the breeding areas^19^. Therefore, spending the non-breeding season in high quality areas and undertaking specific migratory strategies can have important consequences for individual fitness in distant breeding areas^4^.

Reproductive success is also affected by local factors during the breeding season, such as weather conditions or food availability. Particularly for income breeders that use local resources for egg production, unfavourable weather conditions upon arrival may limit food availability and delay egg-laying^21^. In addition, food shortage mediated by low temperatures during chick rearing affects body condition of offspring and consequently depress annual productivity^22^. Adverse conditions at breeding sites may therefore counteract potentially positive carry-over effects incurred at the non-breeding residency sites and during spring migration^23^, or even exacerbate negative carry-over effects between seasons^24^.

In this study, we use geolocators to explore migration performance and its association with breeding phenology and productivity, in European bee-eaters (*Merops apiaster,* hereafter bee-eater) in the Iberian Peninsula. Although the breeding biology of the European bee-eater has been well studied^25–30^, little information on the spatio-temporal distribution of the species is currently available (but see Refs.^31–33^) and the potential effects of migratory performance on productivity have never been explored for this species. The European bee-eater is a long-distance migratory insectivore that breeds in open natural and agricultural areas in the Palearctic and spends the non-breeding season south of Sahara^34^. Bee-eaters are monogamous and both sexes actively contribute to nest (re)construction which takes approximately 10–20 days. Females lay the eggs in 1 to 2 days intervals and nestlings hatch after ca. 20 days. Both parents feed the young until fledging at ca. 30 days of age^34^. Ringing and recovery data have been widely used to track individuals across their migratory range and although bee-eater recoveries have increased, long-distance recoveries are relatively rare (see Ref.^35^). More recently, light-level geolocators unravelled the migratory patterns and non-breeding areas used by five bee-eaters from the Iberian population, which overwintered in West Africa^33^. However, of these, only three were compete tracks. Here, we use a larger dataset to (1) establish a more complete picture of migratory performance and non-breeding distribution of bee-eaters breeding in Iberia, and (2) investigate variation in travel duration and travel speed between seasons. Finally, we (3) explore whether non-breeding latitude, non-breeding departure, in-flight duration and in-flight speed determine arrival date to the breeding areas and (4) if timing of arrival to the breeding areas influences laying dates and productivity, at the individual level. We predict that if breeding early is advantageous, then travel duration and speed will be shorter and faster during spring migration than autumn migration, due to a higher pressure to arrive earlier in the breeding grounds. We also expect that non-breeding latitude, departure time, in-flight duration and speed influence arrival date to the breeding areas. Specifically, bee-eaters occupying more northerly non-breeding areas should arrive earlier, as well as birds departing earlier or spending less time in-flight. As a result, we expect that bee-eaters arriving earlier also start laying eggs earlier, with earlier laying bee-eaters experiencing higher productivity.

## Results

### Non-breeding distribution and migration timings

**Figure 1:**
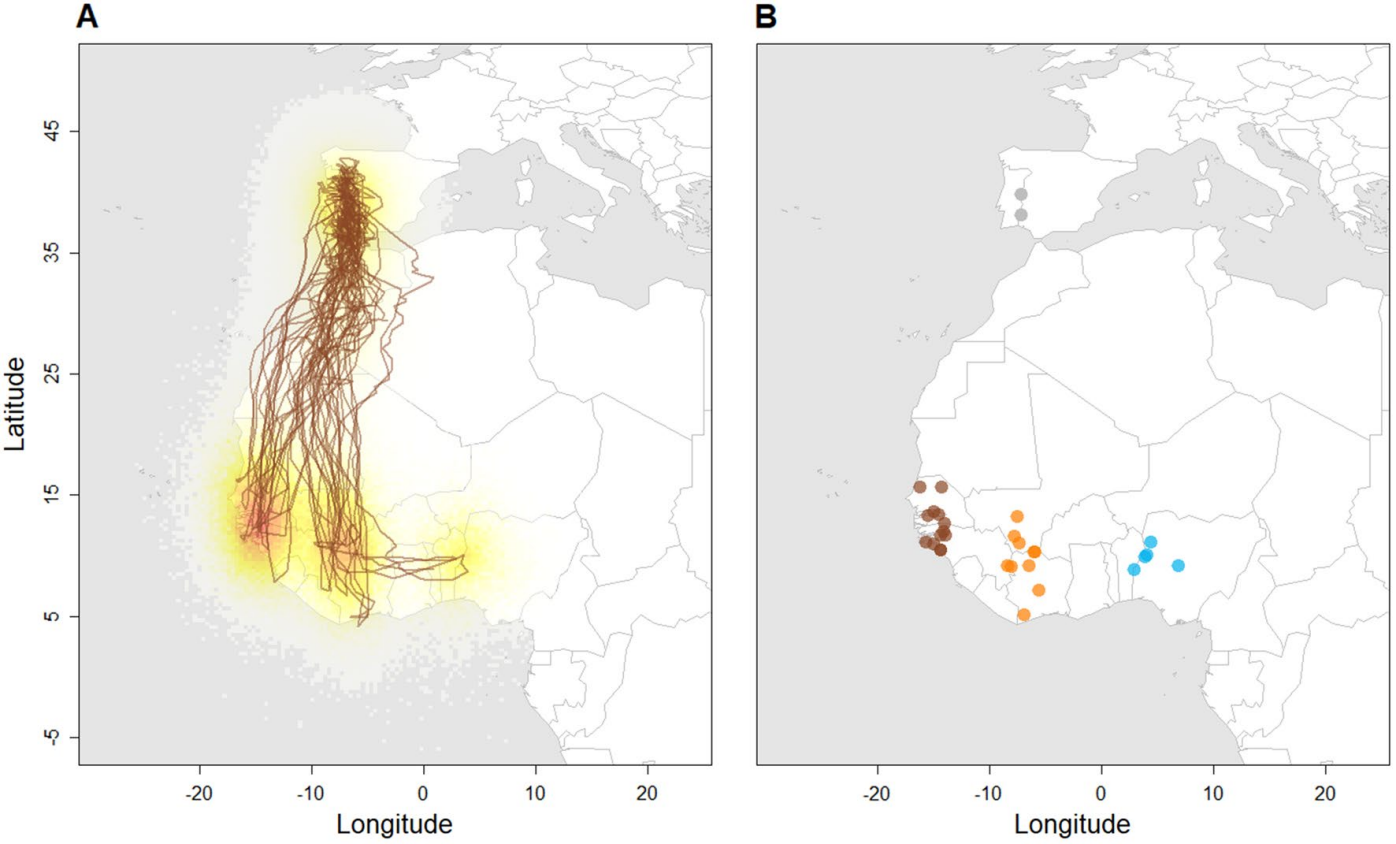
(**A**) Modelled tracks of Iberian bee-eaters with complete annual tracking (n = 25) and the non-breeding ranges of all tracked birds (n = 28). Brown lines represent the median positions and coloured areas represent the 95% probability distributions of location estimates. (**B**) Median position of the breeding (grey) and the non-breeding areas (brown: Senegal/Guinea-Bissau, orange: Mali/Ivory Coast and blue: Benin/Nigeria) of all tracked Iberian bee-eaters (n = 28). Figure was generated in R v.3.4.3 (https://www.r-project.org/).

All tracked bee-eaters from Iberia spent the non-breeding season in West Africa. Most (46.4%) migrated to Senegal and Guinea-Bissau (migration distance: 3009 ± 248 km; mean ± sd; n = 13); 35.7% to the region of Mali/Ivory Coast (migration distance: 3268 ± 316 km; n = 10) and the remaining 17.9% to Benin and Nigeria (migration distance: 3532 ± 109 km; n = 5; Fig. 1). The overall mean migration distance between breeding and non-breeding areas was 3195 ± 319 km.

Bee-eaters departed from the breeding sites between 26 July and 31 August (13 August ± 9.8 days; mean ± sd) and arrived at their non-breeding sites between 1 and 25 September (10 September ± 8.4 days; Table 1). Furthermore, 52% of the birds had a stopover during autumn migration with a mean duration of 11.1 ± 6.8 days (n = 13). During the non-breeding period, bee-eaters spent a mean of 172.6 ± 16.9 days in the main non-breeding areas. Four birds used more than a single residency site and spent a mean of 159.3 ± 26.3 days and 17.0 ± 1.2 days in the main and secondary non-breeding area, respectively. Birds departed from the non-breeding areas between 8 March and 2 April (19 March ± 7.4 days) and arrived in the breeding areas between 27 March and 23 April (7 April ± 7.6 days; Table 1). Half of the birds stopped over once during spring migration (mean stopover duration: 9.5 days ± 4.6 days; n = 11). Interestingly, bee-eaters spending the non-breeding period in the regions Senegal/Guinea-Bissau and Mali/Ivory Coast departed (Senegal: 20 March ± 6 days; Mali: 15 March ± 7 days) and arrived (Senegal: 7 April ± 7 days; Mali: 5 April ± 9 days) earlier, than the two tracked individuals that spent the non-breeding season in Benin/Nigeria, which departed (22^nd^ and 31^st^ March) and arrived considerably later (22^nd^ and 23^rd^ of April).

**Table 1:** Migration parameters for Iberian bee-eaters tracked in 2015–2016 (n = 2), 2016–2017 (n = 22) and 2017–2018 (n = 1). Travel duration (days), travel speed (km/day), in-flight duration (days) and in-flight speed (km/day) refer to migratory periods. Note that precision on exact day of the year may be affected by difficulty in assessing it from geolocator data.

Autumn	Mean	SD	Min	Max
Departure date	13 Aug	9.8	26 Jul	31 Aug
Arrival date	10 Sep	8.4	1 Sep	25 Sep
Travel duration (days)	28.7	12.9	9.0	58.0
Travel speed (km/day)	136.6	70.5	45.7	320.0
In-flight duration (days)	23.5	10.6	9.0	48.5
In-flight speed (km/day)	168.9	86.7	54.7	351.5
**Spring**				
Departure date	19 Mar	7.4	8 Mar	2 Apr
Arrival date	7 Apr	7.6	27 Mar	23 Apr
Travel duration (days)	18.8	7.1	10.0	35.0
Travel speed (km/day)	186.7	63.0	95.4	321.8
In-flight duration (days)	14.3	5.5	6.5	29.5
In-flight speed (km/day)	243.7	85.3	119.8	513.8

**Figure 2:**
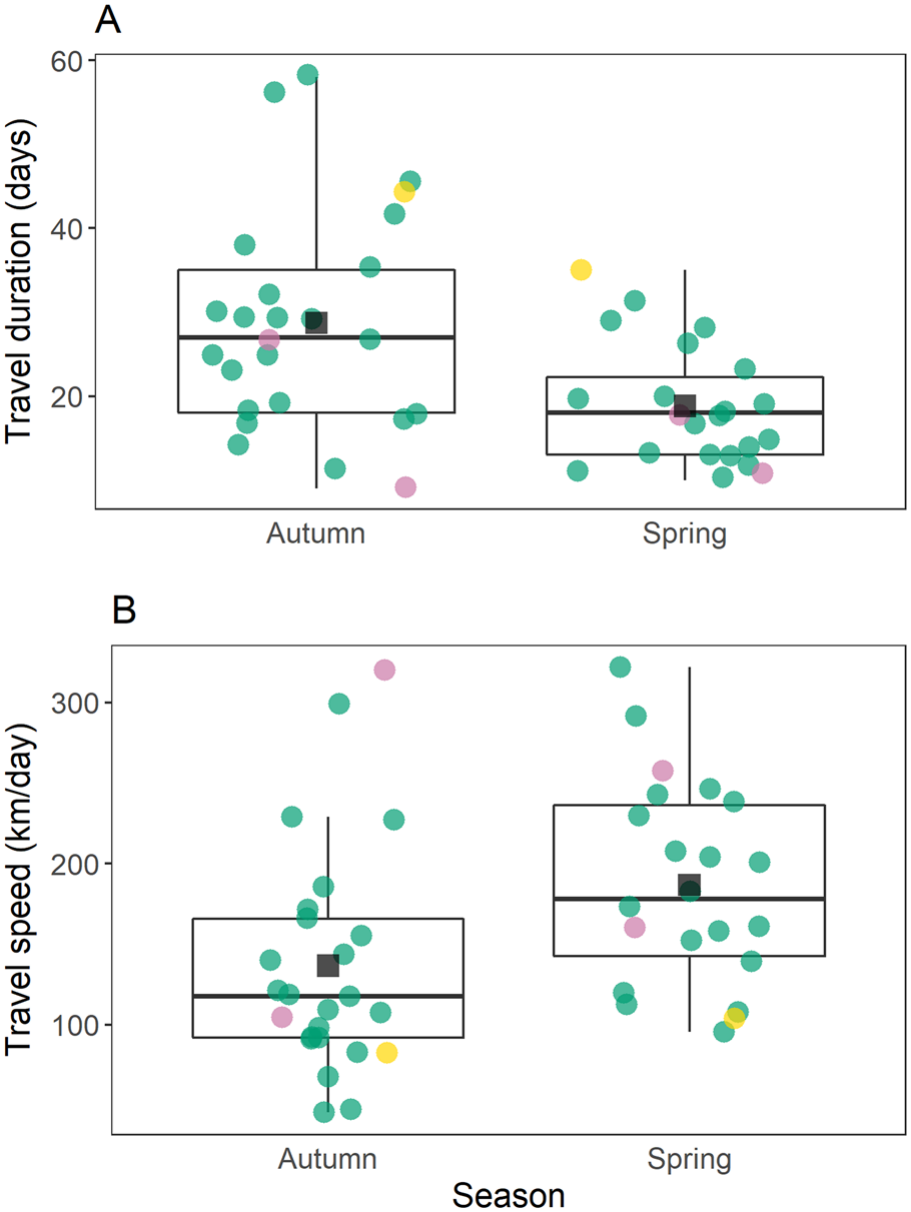
Travel duration (**A**) and travel speed (**B**) of Iberian bee-eaters during autumn and spring migration. Boxes show the median and 25–75% quartiles, whiskers extend up to 1.5 times the inter quartile range from the hinge. Black squares show the mean. Points are coloured by recapture year (2016: pink, 2017: green, 2018: yellow). Figure was generated in R v.3.4.3 (https://www.r-project.org/).

In spring, travel duration was shorter by 9.9 days on average (W = 404.5, *p* = 0.005; Fig. 2A) than in autumn, and travel speed was higher by 50 km/h (W = 144.0, *p* = 0.004; Fig. 2B).

### Drivers of breeding arrival

**Figure 3:**
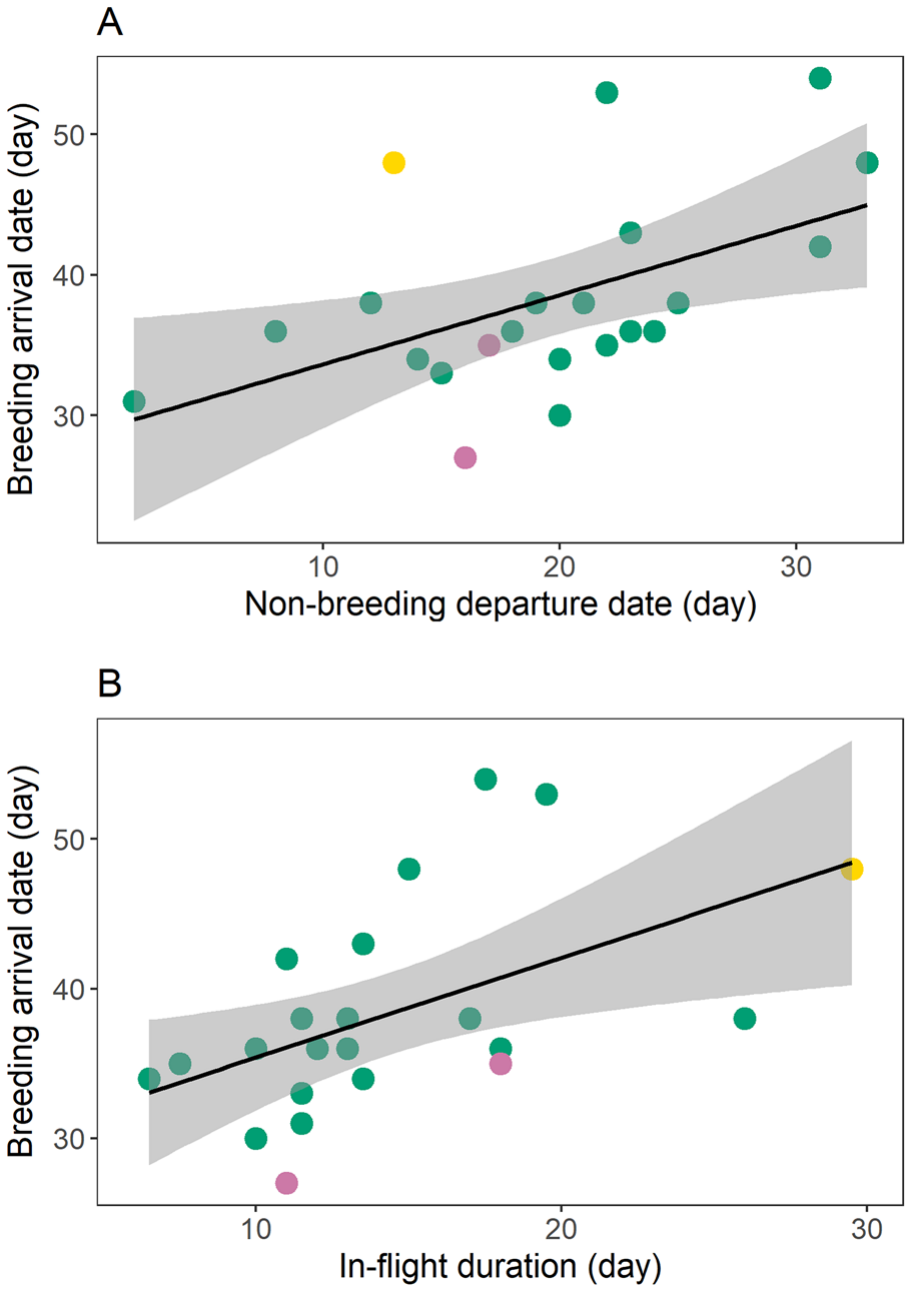
Variation in: (**A**) breeding arrival date and non-breeding departure and (**B**) breeding arrival and spring in-flight duration of tracked Iberian bee-eaters. Dates are shown in number of days from the 1^st^ March, and linear regression lines are drawn with grey shaded area representing 95% confidence interval. Points are coloured by recapture year (2016: pink, 2017: green, 2018: yellow). Figure was generated in R v.3.4.3 (https://www.r-project.org/).

**Figure 4:**
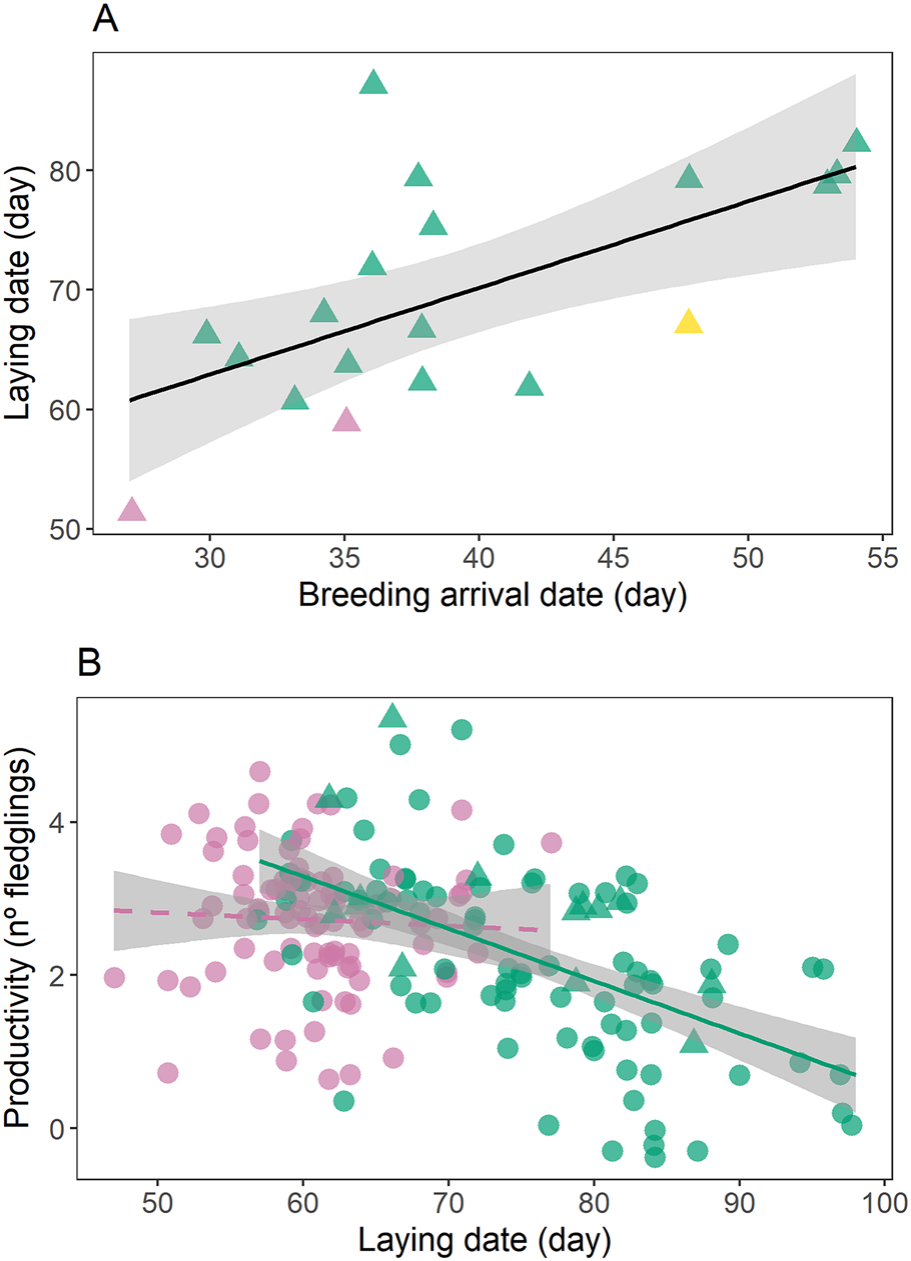
Variation in: (**A**) arrival date at the breeding area and laying dates of tracked Iberian bee-eaters. Pink, green and yellow show birds recaptured in 2016 (n = 2), 2017 (n = 16) and 2018 (n = 1), respectively; and (**B**) laying date and productivity per nest in 2016 (n = 87, pink) and 2017 (n = 96, green). Nests of tracked birds are represented with triangles and other nests with circles. In both plots, dates are shown in number of days from the1^st^ of March and linear regression lines are drawn with grey shaded area representing 95% confidence interval (solid line indicates significant relationships). Figure was generated in R v.3.4.3 (https://www.r-project.org/).

Departure from the non-breeding areas and in-flight duration were the best predictors for explaining the variation in arrival date at the breeding sites (AICc = 137.18, Table S1). Departure date from the non-breeding area and in-flight duration were positively related with breeding arrival date. More specifically, birds departing earlier from the non-breeding areas, or having shorter in-flight duration, tended to arrive earlier to the breeding areas (Table 2, Fig. 3A,B). In-flight duration (Spearman correlation test: S = 1799.1, p = 0.944, rho = -0.01), and stopover length (Spearman correlation test: S = 322.34, p = 0.149, rho = -0.465) were not correlated with non-breeding departure date. Arrival at the breeding areas positively affected laying dates, as birds arriving first laid their eggs earlier (laying date = 0.724 × arrival date + 41.211; t = 3.291; *p* = 0.004; r^2^ = 0.35; n = 19; Fig. 4A).

**Table 2:** Results of the top ranked linear model (Non-breeding departure + in-flight duration) predicting breeding arrival time.

	Estimate	SE	t	*p*
Intercept	16.70	4.11	4.06	<0.001
Non-breeding departure	0.55	0.13	4.01	< 0.001
In-flight duration	0.75	0.18	4.05	< 0.001

### Relationship between laying date and productivity

Annual productivity was significantly different between years, with birds in 2016 producing overall more fledglings (mean = 2.72 ± 0.88) than in 2017 (mean = 2.23 ± 1.85; Table 3, Fig. 4B). The results show a negative relationship between timing of laying and productivity in 2017 (Table 3, Fig. 4B), as the number of fledglings decreased along the season. However, this was not the case in 2016, when productivity remained constant throughout (Table 3, Fig. 4B). We also found a negative relationship between timing of laying and productivity, when considering only the tracked bee-eaters from both years (productivity =  − 0.058 × laying date + 7.145; t =  − 2.911; *p* = 0.010; r^2^ = 0.32; n = 17). It was therefore possible to establish, at the individual level, a positive effect of arrival date on laying date and a negative effect of laying date on productivity, therefore highlighting the important effects of early arrival on productivity.

**Table 3:** Results of the linear models testing the relationship between laying date and productivity in 2016, 2017 and in both years combined.

		Estimate	SE	t	*p*
2016 + 2017	Intercept	3.248	1.148	2.827	0.005
	Laying date	− 0.008	0.018	− 0.458	0.647
	Year	4.141	1.359	3.047	**0.002**
	Laying date × year	− 0.059	0.021	− 2.823	**0.005**
2016	Intercept	5.248	1.094	4.797	< 0.001
	Laying date	− 0.008	0.017	− 0.481	0.632
2017	Intercept	7.389	0.756	9.771	< 0.001
	Laying date	− 0.068	0.009	− 6.869	** < 0.001**

Bold *p*-values indicate statistical significance, *p* ≤0.05.

## Discussion

This study shows that earlier departure from the non-breeding areas and shorter in-flight duration likely contributed to earlier arrival in the breeding grounds. This can have important consequences for breeding success and ultimately demographic rates, as individuals that arrived earlier had higher probability of laying earlier and producing more offspring overall. However, this pattern is not consistent across years, indicating that local factors in the breeding areas also play an important role in determining annual productivity.

Bee-eaters from Iberia spent the non-breeding season in West Africa between Senegal and Nigeria, confirming earlier findings. While most Iberian bee-eaters stayed between Senegal and Guinea-Bissau, the other two non-breeding areas had decreasing proportions of tagged birds with increasing distance from the breeding area (Guinea-Bissau n = 13; Mali/Ivory Coast n = 10 and Benin/Nigeria n = 5). Interestingly, all Iberian bee-eaters migrated traveling along the western coast of Africa. Individuals occupying more easterly longitudes (i.e. Benin/Nigeria) did not take a more direct inland route across the Sahara, as has previously been observed with bee-eaters from Germany^33^. Traveling in a more inland route would shorten the travel distance between breeding and non-breeding areas by ca. 15%, but would also increase the crossing over the Sahara desert and thus the probability of facing less favourable conditions due to a potentially low availability of flying insects (bee-eaters main prey).

We found that Iberian bee-eaters have higher travel speed and shorter travel duration in spring compared to autumn. This is common in many migratory species^12,36,37^ since early arrival at the breeding areas is often linked to higher productivity, while early arrival to non-breeding areas may not be so tightly linked to apparent benefits (but see Ref.^38^). Environmental conditions during migration may also influence arrival to the breeding grounds. For example, thermal uplift may provide considerable subsidy for migration^39^, but seasonal differences in thermal availability remain to be explored. Indeed, for bee-eaters breeding in Iberia, early arrival promotes higher productivity, although this seems to also be modulated by conditions experienced in the breeding area (see below).

For several species, non-breeding departure date is a strong predictor of arrival date at the breeding site^14,40,41^, but differences in the duration of migratory flights can also contribute to the variation in arrival^15,16^. Our results show that bee-eaters departing earlier or spending less time in-flight arrive earlier to the breeding areas. Given that in-flight duration was not correlated with non-breeding departure, departure from the non-breeding areas is likely the main factor influencing arrival order to the breeding areas in this population. Faster migration by late departing individuals was reported for several species of *Sylvia* warblers, but only during autumn migration^42^; while in Great reed warblers (*Acrocephalus arundinaceus*), later departing individuals do not seem to compensate for their delays in spring^40^. Late departing Collared flycatchers (*Ficedula albicollis*) also show higher migration travel speed, but they seem able to partially catch up with early departing individuals and arrive at the breeding destination at a similar time^43^. It is possible that bee-eaters can only reduce in-flight duration to a certain limit since they are known to rely, at least partially, on a fly-and-forage strategy^34^. On the contrary, flycatchers, like many passerines, are known to fuel before migration and therefore may be able to maximize travel speed^43^, for example in a final sprint to finish migration^44^.

Bee-eaters departed from the non-breeding areas over a relatively long period (8 Mar–2 Apr). The variation in non-breeding departure, and consequently breeding arrival dates, may have been be influenced by several factors, including local weather conditions, individual body condition and the group dynamics characteristic of bee-eaters. This species is known to form groups that can remain together during one or several stages of the annual cycle or even throughout a complete annual cycle^32^. These associations might influence the timing of their activity, for example timing of departure. The late departure of the two individuals that spent the non-breeding season in Nigeria could be explained by a late departure of the social group these individuals were part of, as in this area they overlap with bee-eaters traveling to breeding sites in Germany, which are known to depart later (although this includes bee-eaters spending the non-breeding season further south^33^). Late arrival could also be due to the longer travel distance from this location. However, the distance travelled by birds spending the non-breeding season in Senegal/Guinea Bissau or Mali/Ivory Coast differs only by ca. 260 km and by ca. 265 km respectively, from bee-eaters spending the non-breeding period in Nigeria, further suggesting that other factors like group dynamics or conditions experienced during non-breeding period, rather than distance, may play a role in determining arrival dates in the breeding areas.

Bee-eaters in spring travelled on average 18.8 days (range 10–35 days) at a mean speed of 186 km/day (range 95–321 km/day). Although travel speed varied between individuals, it was overall low compared to other aerial insectivores migrating between Europe and Africa like Common swifts (*Apus apus*, 326 km/day^37^) or Barn swallows (approximately 320 km/day^45^). Individuals that departed earlier were possibly less constrained by time and could have had more time to forage during migration, while late departing individuals may be trying to compensate for the delay, but the lack of correlation between non-breeding departure and stop-over length suggests this is unlikely. In any case, conditions during migration are also an important factor influencing migratory rates as reported in Red-backed shrikes (*Lanius collurio*) and Thrush nightingales (*Luscinia luscinia*), which showed delayed arrival at the breeding grounds after experiencing a severe drought (at stopover sites) during migration^46^.

We report an overall positive relationship between arrival and laying dates for Iberian bee-eaters. Tracked individuals arriving and laying earlier also had higher productivity compared to those that laid their eggs later, in the 2017 breeding season. Although the mechanism by which early arrival leads to earlier laying in this population is unknown, early-arriving birds are able to start laying their clutches earlier^47^ possibly because they quickly find suitable nesting sites, which are nevertheless not limiting in the studied colonies (several unoccupied sand walls and empty nests were recorded each season, JSC *pers. obs.*). However, in our study, 65% of the variation observed remained unexplained and laying dates are likely influenced by other factors besides arrival date.

Despite the lack of an overall effect of laying date on productivity, this relationship was in fact year specific. In 2016, laying was overall earlier in the season and productivity remained constant throughout. Conversely, while early laying birds in 2017 had similar productivity compared to birds in 2016, the number of fledglings decreased seasonally. The first birds recorded on camera traps set on each colony arrived at similar dates in both years (2016: 30 March; 2017: 31 March), indicating that earlier laying in 2016 was unlikely due to individuals arriving much earlier than in 2017. Also, and despite the low sample size, in years other than 2017 the arrival date of tracked individuals does not reveal considerable differences between years (Fig. 4A). This suggests that poorer breeding conditions, for instance low food availability mediated by unfavourable weather conditions^22^ at the onset of laying in 2017, could have delayed egg production despite the very early birds having arrived at similar time in both years. Additionally, productivity was on average higher in 2016 compared to 2017, suggesting that in years when breeding conditions are favourable, early and late breeders will produce high and similar numbers of fledglings. But when facing unfavourable breeding conditions, only early laying individuals will experience high productivity. Indeed, conditions at the breeding grounds can have a larger effect on breeding phenology and success, than carry-over effects from previous seasons^21^. In our study area, June of 2017 was the 3^rd^ hottest and driest since 1931 with a heat wave occurring between 10^th^ and 21^st^ June (daily maximum temperatures above 35ºC for 9 days^48^). As many broods hatched close to and during this heat wave (mean hatching date: 4^th^ June, range: 14^th^ May–26^th^ June) it is thus possible that the exceptionally high temperatures negatively affected breeding conditions of late breeders in 2017. Overall, insects are known to make behavioural adjustments to regulate body condition within an optimum range^49^, and above a temperature threshold, they possibly search for cooler microclimates, retreating into the shade. As bee-eaters feed on aerial insects reduced prey availability during the heat wave could have led to a decrease in provisioning rates. Alternatively, adult bee-eaters could have decreased nest attendance and chick provisioning due to physiological costs of water loss and heat stress resulting from intense activity. Although the effect of high temperatures on productivity of birds have been mainly studied in arid and semi-arid habitats^50,51^, it is possible that species breeding in the Mediterranean, where extreme heat events are becoming more frequent^52^, are increasingly experiencing similar pressures. In addition, predation could have been higher in 2017 compared to 2016 due to an increase in the number of predators in the surroundings, or by an indirect effect of the prolonged high temperatures and associated depletion of alternative food sources for predators^53^. In any case, further work such as identifying factors influencing bee-eater phenology during non-breeding (migration timing) and breeding seasons (laying dates) in multiple years, as well as assessing nestlings’ body condition and its potential drivers, will provide insights into the exact processes underlying these inter-annual differences in productivity of bee-eaters breeding in Iberia.

## Methods

Between 2015 and 2018, we used geolocation by light to track adult bee-eaters between their breeding colonies in Portugal (38.1°N, − 7.0°E and 39.8°N, − 7.1°E) and their non-breeding areas. Bee-eaters were captured in the nest burrows with walk-in traps during the nestling provisioning period. Each year, we equipped 60 bee-eaters with geolocators (total 180; SOI-GDL1/GDL3-PAM; Swiss Ornithological Institute) using a leg-loop harness made from Silicone or cord material (Table 4). The average mass of the geolocators, including harnesses, was 1.33–1.45 g, comprising less than 3% of bee-eater body mass. The annual recapture rate of birds with geolocators was 6.6% in 2016, 36.7% in 2017 and 1.6% in 2018. Additionally, we ringed birds with a metal ring only (2015: 57, 2016: 213, 2017: 239) from which the recapture rates were 24.2% in 2016, 32.1% in 2017 and 13.2% in 2018. We successfully tracked 28 birds with geolocators resulting in 22 full annual tracks, 3 incomplete tracks (autumn migration only) and 28 non-breeding sites (Table 4). Sampling effort did not differ between years, with more than 90% of the individuals in each colony captured each year. Recapture rates for the species are low^33^ and its inter-annual variability is possibly influenced by breeding site dispersal^31^. Birds were captured and tagged under permissions issued by the relevant national authority (Instituto da Conservação da Natureza e das Florestas; ringing permits number 1/2015, 102/2016, 106/2017 and 1/2018), which issues such licences within the framework of a specific study and ensures ethic standard when handling and marking birds, given the methods used (which are explicitly stated in the license). As part of a PhD thesis, all activities reported in this study were approved by the Scientific Council of the University of Aveiro. All methods were carried out in accordance with relevant guidelines and regulations.

**Table 4:** Number of Iberian bee-eaters equipped with geolocators and the resulting records for autumn and spring migration and non-breeding area location; (n total (females /males).

Year	Type	Deployed	Total recovered	Autumn	Spring	Non-breeding
2015/2016	GDL1	60	5 (4 /1)	2 (1/1)	2 (1/1)	5 (4/1)
2016/2017	GDL3	60	22 (13/9)	22 (13/9)	19 (13/6)	22 (13/9)
2017/2018	GDL3	60	1 (male)	1	1	1

### Productivity and laying dates

In order to determine productivity (total number of fledglings per nest), we visited the colonies weekly and recorded the number of pre-fledging nestlings during the third week of development using a “burrowscope”. Typically, bee-eaters lay one clutch per year^34^ and renesting after failure was not recorded in the studied colonies (JSC pers. obs.). Bee-eaters take 30 days to fledge and it was assumed that the number of nestlings recorded in the third week of development reflected the total number of fledgling’s produced. We estimated hatching date of each brood using a photographic guide for age determination of bee-eater nestlings^54^. Laying dates were back-calculated by subtracting the incubation period of 20 days to the hatching date of the first egg. We determined the laying date and productivity of 87 nests in 2016 (including two of the tracked birds in 2015–2016) and 96 nests in 2017 (including of 19 tracked birds in 2016–2017; although in two of these cases productivity was not possible to quantify due to the sinuous shape of nest tunnel and chamber, which made it impossible to clearly observe the nestlings). In 2018 it was only possible to determine the laying date of the single tracked bee-eater.

### Geolocation analysis

Geolocators recorded light intensity at 2 (GDL1) and 5 min (GDL3) intervals. After log transforming the light intensity data, we used a threshold method to identify sunrise and sunset events (using a threshold of 3, except for two birds that required threshold of 8 and − 5) with R package *TwGeos*^55^. This step allowed the detection of errors on twilight events and to be manually corrected when necessary. Twilight events can be influenced by shading (e.g. due to clouds or foliage) potentially resulting in shorter days and affecting location estimates^56^. Therefore, we quantified the error distribution of sunrise/sunset times by using twilights from a known location (i.e. the breeding colony). More specifically, we used the recordings from a day after the geolocator was fitted on the bird until before the start of migration (range: 40–50 days) and calibrated the data by fitting an error distribution to the sunrise and sunset data, which was later used by SGAT (see below). We then plotted the estimated latitude of the bird over time with the estimated zenith from breeding calibration; if the latitude during stationary periods was not flat over time (i.e. lowest variance in latitude estimates) we used a Hill-Ekstrom calibration to adjust the estimated zenith^56^. For Hill-Ekstrom calibration we defined a period when the bird was in the non-breeding area (usually from the first half of October to beginning of March).

We used a group model in the R package *SGAT*^57^ to estimate geographic positions. The group model uses known stationary periods to estimate a single location from multiple twilight events. This increases the accuracy of the location estimate^58^. First, we used the *changelight* function from the package *Geolight*^59^ to separate periods of residency from periods of movement, based on changes in sunrise and sunset times. The function uses the difference in day length to estimate movement periods given a change probability *q*. Due to the high probability of errors when determining stationary periods of short duration, the high sensitivity of *changelight* function to data quality and specificity to each device^59^, and given the variation in data quality among individuals, we selected different parameters for each individual. More specifically, we defined stationary periods ranging from 4 to 8 days and defined a probability of change from 0.80 to 0.95. The identified stationary periods were then merged together using function *mergeSites* from *Geolight* package^59^.

*SGAT* was used to find the best possible fit to the data and increase the precision of the estimated locations. SGAT uses a Bayesian framework incorporating prior information, such as the previously defined stopover periods, twilight error distribution (from the calibration), flight speed distribution and a spatial probability mask (to ensure that when a bird stops, it is less likely to do so in the sea). Markov Chain Monte Carlo simulations then allow the model to simulate the geographic probability distribution of each location. We fixed the first and last location to the known capture and recapture locations except for cases when the sensor stopped logging before the recapture date (n = 6). We first ran a modified Gamma model (relaxed assumptions) for 1000 iterations to initiate the model, before tuning the model with final assumptions/priors (three runs with 300 iterations). Finally, the model was run for 2000 iterations to ensure convergence. For the datasets that only recorded light during a short period of the non-breeding season we ran SGAT only during the period when light was recorded.

We defined stopover sites as stationary periods located north of the Sahara or south of the Sahara lasting up to 14 days. We defined non-breeding residence sites as a stationary period longer than 14 days occurring south of the Sahara. For birds that used more than one residency site, we defined the main non-breeding site as the longest stationary period south of the Sahara and used that site to extract non-breeding area latitude and longitude.

### Migration distance, travel duration and travel speed

We calculated migration distance considering the orthodromic (great circle) line between the breeding and the non-breeding areas (in km). For birds with more than one non-breeding site (n = 4; individuals with two residency sites) we considered the first residency site (for autumn migration) or the last one (for spring migration). To define arrival to and departure from the non-breeding areas for birds with more than one non-breeding site (as Julian dates), we considered the first residency site and the last one, respectively. We calculated seasonal travel duration as the number of days between departure from breeding/non-breeding sites and arrival at final seasonal destination. Travel speed of migratory movements was defined as the total distance (in kilometres) divided by travel duration. Given that spatial data inferred from geolocator does not allow determining when pre-departure fuelling was initiated, we excluded stopover periods to estimate in-flight duration and in-flight speed on active migration. We therefore calculated seasonal in-flight duration as the number of days between departure from breeding/non-breeding sites and arrival at final seasonal destination excluding stopover duration. In-flight speed was calculated as the total distance divided by in-flight duration. Note that precision on exact day of the year may be affected by difficulty in assessing it from geolocator data. Nevertheless, given all geolocator data underwent the same procedure it is unlikely that a systematic bias may have emerged.

### Statistical analysis

We used Wilcoxon tests to explore seasonal differences in travel duration and travel speed. In order to explore which factors may be influencing arrival date to the breeding areas we constructed a linear model with non-breeding latitude, spring in-flight duration, spring in-flight speed and departure date from the non-breeding areas as main predictors. We ran full, reduced, and null models that were ranked according to Akaike’s Information Criterion for small sample sizes (AICc). Differences between models were assessed by the difference in AICc scores (∆AICc; R-package AICcmodavg^60^) from the model with the smallest AICc (Table S1). Since in-flight speed and in-flight duration were strongly correlated (Spearman correlation test: S = 3447.3, *p* < 0.001, rho =  − 0.94) both predictors were never included in the same model (all other variables were not strongly correlated: rho<0.3). Due to the low number of samples in 2015 and 2018, it was not possible to compare migration timings between years. Additionally, we constructed a linear regression model to test if arrival date to breeding area influences laying date at the individual level.

In order to test if productivity is influenced by laying date, we first performed a general linear model (gaussian error structure and identity function) using the complete dataset (i.e. nests from tracked and non-tracked birds) and both years combined (i.e. 2016 and 2017), having laying date, year and its interaction as main predictors. As both year and the interaction term were significant, suggesting that the laying date effects on productivity differed between years, we performed a linear regression model for each year separately, using only laying date as main predictor. Finally, to investigate if the links between arrival, laying date and productivity were also apparent at the individual level, we ran a linear regression model using only the tracked individuals, having lay date as predictor of productivity. We did not find statistical differences in productivity and laying dates between both colonies and all the nests considered in this analysis did not present any signs of predation. Linear model assumptions were confirmed by graphical inspection of standardized residuals and by plotting residuals against fitted values. All statistical analyses were performed in R 3.4.3^61^.

## Supplementary Information

Supplementary Information.

## Data Availability

Tracking data are available upon request from Movebank online database (https://www.movebank.org/, study IDs: 1416257300, 1416270261, 1416275401). Datasets of breeding phenology and productivity are available from the corresponding author on reasonable request.
