# Supplementary material for: Linking migratory performance to breeding phenology and productivity in an Afro-Palearctic long-distance migrant
Source: Sci Rep. 2021 Dec 1;11:23258. doi: 10.1038/s41598-021-01734-0 (PMC8636482; doi:10.1038/s41598-021-01734-0)
Supplement: Supplementary file 1 — Supplementary Information. [file 41598_2021_1734_MOESM1_ESM.pdf]

Supplementary Information

Linking migratory performance to breeding phenology and productivity in an Afro-Palearctic long-distance migrant

Authors: Joana S. Costa[1*], Steffen Hahn[2], Pedro M. Araújo[3,4], Kiran L. Dhanjal-Adams[2,5,6], Afonso D. Rocha[1] & José A. Alves[1,7]

[1] CESAM - Centre for Environmental and Marine Studies, Department of Biology, University of Aveiro, Portugal

[2] Department of Bird Migration, Swiss Ornithological Institute, Sempach, Switzerland.

[3] CIBIO/InBIO, Centro de Investigação em Biodiversidade e Recursos Genéticos, Campus Agrário de Vairão, Universidade do Porto, Vairão, Portugal.

[4] MARE – Marine and Environmental Sciences Centre, Department of Life Sciences, University of Coimbra, Coimbra, Portugal.

[5] Centre for the Advanced Study of Collective Behaviour, University of Konstanz, Konstanz, Germany.

[6] Ecology of Animal Societies, Max Planck Institute of Animal Behavior, Konstanz, Germany

[7] South Iceland Research Centre, University of Iceland, Laugarvatn, Iceland.

*corresponding author: joana.santcosta@gmail.com

Supplementary Table

Table S1. Ranking of candidate models predicting arrival date to the breeding area. AICc = Akaike information criterion for small sample size; ΔAICc = difference between AICc of the best model and reduced model; AICcw = Akaike model weights; loglik = log-likelihood; k = number of model parameters. Model with the lowest ΔAICc is shown in bold.

**Model**	**AICc**	**ΔAICc**	**AICc*w***	**loglik**	**k**
NB departure + in-flight duration	**137.18**	**0.00**	**0.74**	**-63.42**	**4**
NB latitude + NB departure + in-flight duration	139.98	2.79	0.18	-63.11	5
NB latitude + NB departure + in-flight speed	143.10	5.91	0.04	-64.67	5
NB departure + in-flight speed	144.30	7.12	0.02	-66.97	4
NB latitude + NB departure	147.33	10.14	0.00	-68.49	4
In-flight duration	147.69	10.51	0.00	-70.18	3
NB departure	147.89	10.70	0.00	-70.28	3
NB latitude + in-flight duration	149.93	12.74	0.00	-69.79	4
NB latitude + in-flight speed	150.61	13.43	0.00	-70.13	4
In-flight speed	150.95	13.77	0.00	-71.81	3
NB latitude	151.74	14.56	0.00	-72.20	3
Null	152.00	14.81	0.00	-73.68	2
